# Active Community-Based Case Finding of Endemic Leishmaniasis in West Bengal, India

**DOI:** 10.1007/s44197-024-00260-2

**Published:** 2024-06-17

**Authors:** Subhasish Kamal Guha, Ashif Ali Sardar, Amartya Kumar Misra, Pabitra Saha, Anwesha Samanta, Dipankar Maji, Amitabha Mandal, Punita Saha, Supriya Halder, Kabiul Akhter Ali, Sibajyoti Karmakar, Dipendra Sharma, Ardhendu Kumar Maji

**Affiliations:** 1 Calcutta School of Tropical Medicine, 108, C. R. Avenue, Kolkata, West Bengal India; 2 Department of Microbiology, Calcutta School of Tropical Medicine, 108, C. R. Avenue, Kolkata, West Bengal India; 3 Department of Tropical Medicine, Calcutta School of Tropical Medicine, 108, C. R. Avenue, Kolkata, West Bengal India; 4 Present Address: Department of Zoology, P. R. Thakur Government College, Thakurnagar, North 24 Parganas, West Bengal India; 5 Department of Health and Family Welfare, Government of West Bengal, Swasthya Bhavan, Salt Lake City, Kolkata, West Bengal India; 6 Office of the Chief Medical Officer of Health, Public Health Wing, Malda Medical College Campus, Malda, West Bengal India; 7 R. N. Ray Rural Hospital, Bulbulchandi, Habibpur, Malda, West Bengal India; 8 Office of the Chief Medical Officer of Health, Public Health Wing, Raiganj, Uttar Dinajpur, West Bengal India; 9 Office of the Chief Medical Officer of Health, Public Health Wing, Siliguri, Darjeeling, West Bengal India

**Keywords:** Hidden pathogen pool, Resurgence, Recurrent Post kala azar dermal leishmaniasis, Recurrent VL

## Abstract

**Introduction:**

The ongoing visceral leishmaniasis (VL) elimination programme in India is targeting the elimination of the disease VL but not the pathogen. The persistence of hidden parasite pool may initiate a resurgence in suitable conditions. This study dealt with a novel approach to unearth such pathogen pool and their proper management to prevent the resurgence of VL.

**Materials and Methods:**

We deployed a new approach for detection of pathogen pool by following up the VL and post kala-azar dermal leishmaniasis patients treated during the last 10 years along with mass sero-surveillance within a radius of 500 m of recently treated individuals.

**Results:**

We followed up 72.6% (3026/4168) previously treated VL and post kala-azar dermal leishmaniasis patients and diagnosed 42 (1.4%) new and 38 (1.3%) recurrent post kala-azar dermal leishmaniasis. We detected 93 asymptomatic leishmanial infection, 8 VL and 1 post kala-azar dermal leishmaniasis by mass sero-surveillance.

**Conclusion:**

Our three-step process including mapping and follow-up of previously treated cases, mass surveillance within 500 m of radius of known cases, and 6 monthly follow-on clinical and serological screening of asymptomatic cases, enabled detection of previously undetected cases of post kala-azar dermal leishmaniasis and VL. Recurrent post kala-azar dermal leishmaniasis deserves special attention regarding their treatment guideline. Early diagnosis and effective treatment of all leishmaniasis cases will hasten pathogen elimination and prevent resurgence of VL. This may help the policymakers to develop appropriate strategy for elimination of pathogen to prevent resurgence of VL.

## Introduction

Visceral leishmaniasis (VL), also known as kala-azar, is a vector borne neglected tropical disease caused by a protozoa belonging to the genus *Leishmania* and is transmitted by female sandfly of the genus *Phlebotomous* [1]. More than 80 endemic countries in tropical and subtropical regions reported an estimated 50,000–90,000 new VL cases annually. Ten countries i.e., Brazil, China, Ethiopia, India, Iraq, Kenya, Nepal, Somalia, South Sudan, and Sudan reported 95% of the global VL cases [2]. Three adjoining countries of WHO South East Asia region - India, Bangladesh and Nepal account for more than 50% of the global burden, to which India contributes the major share [3]. In India, about 165.4 million population of 54 endemic districts of Bihar, Jharkhand, West Bengal, and Uttar Pradesh are at risk of infection [4]. India reported 818 VL cases in 2022, of which 52 were from West Bengal [4].

India, Bangladesh, and Nepal jointly launched a VL elimination programme in 2005 supported by the World Health Organization (WHO). The initiative was aimed to eliminate VL as a public health problem by 2015 by reducing the annual incidence to less than 1/10,000 population at the sub-district or district level [4, 5]. The deadline was subsequently reset to 2017 and then to 2023 [6]. Nepal and Bangladesh have reached the target in 2013 and 2016 respectively [7, 8]. India is close to the goal except in few hot spot areas with ongoing transmission [9]. Historically, VL epidemics in India occur cyclically at an interval of 10–15 years [10]. In the Indian subcontinent *Leishmania donovani* is the only parasite, *Phlebotomus argentipes* is the sole vector, and man is the only vertebrate host. The average life span of female sandfly is about 12 days [11]. So, the hidden parasite pools persist in humans during inter-epidemic period and serve as the source of pathogens for resurgence of VL. Two forms of leishmaniasis - post kala-azar dermal leishmaniasis and asymptomatic leishmanial infection play a crucial role as such reservoir. Post kala-azar dermal leishmaniasis, a dermal morbidity characterised by macular, maculo-papular, and nodular lesions, usually developed among 15–20% of apparently cured VL patients following months to years of treatment [12]. Individuals, without prior VL, may also develop the condition [12–17]. It has long been considered to act as the only inter-epidemic reservoir of anthroponotic VL, and the existence of a few cases is sufficient to trigger a new epidemic in a given community [12, 15, 18]. Recently xenodiagnosis studies proved that all forms of post kala-azar dermal leishmaniasis are infectious to sandflies [19]. There is no standard definition of asymptomatic leishmanial infection, but is usually diagnosed by a positive serological test, polymerase chain reaction (PCR), or leishmanin skin test [20, 21]. Mathematical modelling suggests that asymptomatic leishmanial infection may serve as a reservoir of parasites which may cause resurgence of VL, although their infectiousness to sandflies is not well established [22]. The rural poor people, commonly affected with leishmaniasis, are not much concerned about the skin lesions of post kala-azar dermal leishmaniasis. Usually, they do not consult the health service provider during early stage and thus maintain the pathogen in the community. Searching of such parasite pool in the community poses a great challenge to the health system.

Researchers conducted several studies to improve the detection of VL at community level by implementing various case search strategies [23, 24]. But no such effort has so far been made for detection of post kala-azar dermal leishmaniasis. This study was aimed to eliminate the hidden parasite pool using a novel approach of enhanced active case-search, along with the ongoing national programme to prevent the resurgence of VL in endemic districts of West Bengal, India.

## Materials and Methods

### Study Areas

**Fig. 1:**
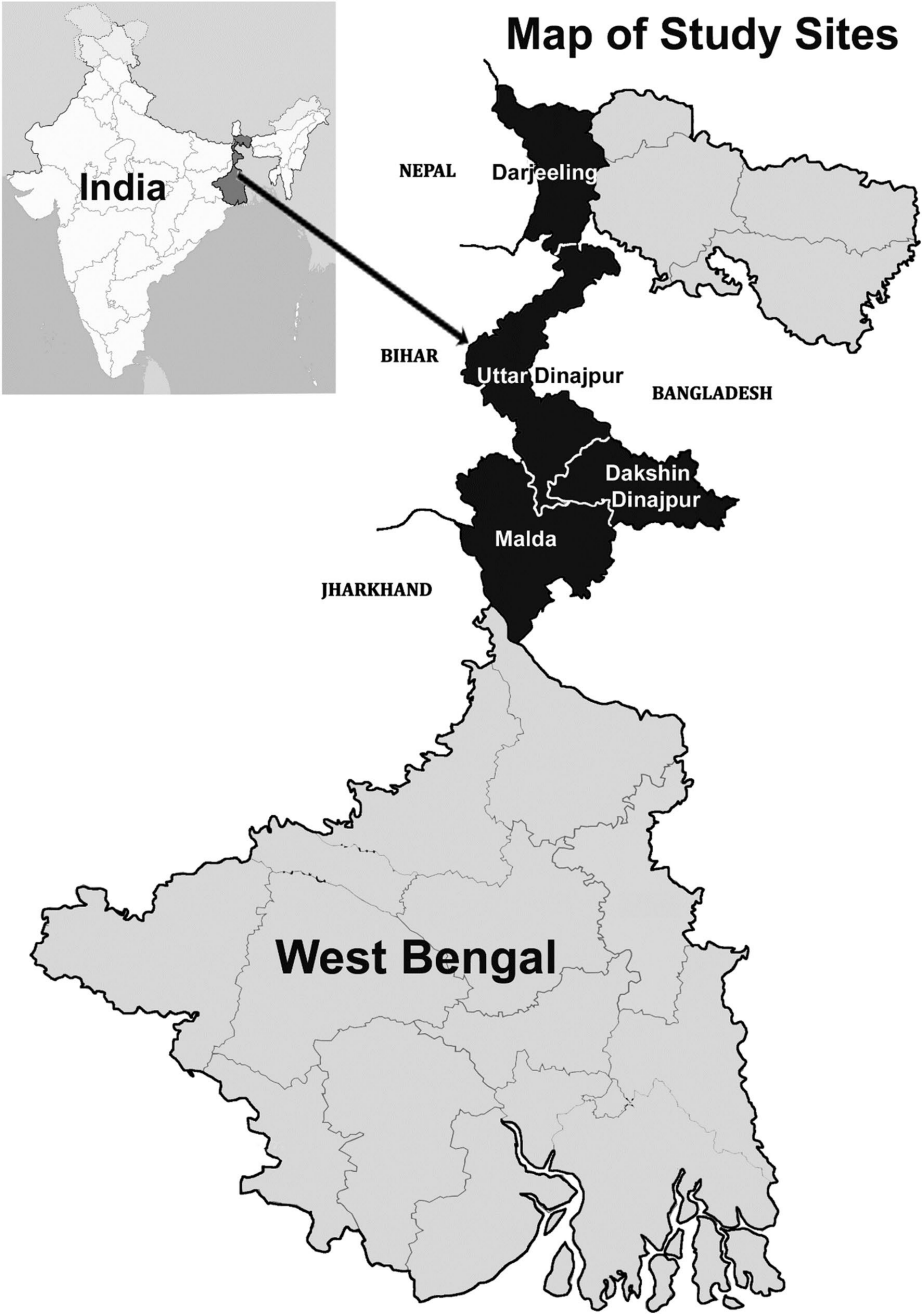
Map showing the study districts of northern West Bengal, India

West Bengal is one of the four provinces of India which is endemic for VL for long time. This study was undertaken in four VL endemic districts of West Bengal - Darjeeling, Uttar Dinajpur, Dakshin Dinajpur and Malda during January, 2022 - March, 2023. All these districts are situated on the northern side of the river Ganges with an international border with Bangladesh in the east and inter-state border with Bihar and Jharkhand (both endemic for VL) in the west. Darjeeling district has an international border with Nepal (in the west) (Fig. 1). All the neighbouring countries are endemic for VL. So, the study areas play an important role in trans-border transmission among three adjoining countries.

### Study Design and Searching of Hidden Pathogen Pool

It was a community based longitudinal study for the detection of hidden parasite pools and their proper treatment for prevention of resurgence of VL. We searched for such pathogen pools using two different ways. First, we adopted a novel approach by clinical and serological follow-up of previously treated VL and post kala-azar dermal leishmaniasis patients as in Indian subcontinent most of the post kala-azar dermal leishmaniasis develop among treated VL subjects [25]. We collected the line-list of VL and post kala-azar dermal leishmaniasis patients treated during last 10 years from the State Health authority and verified with the records of respective districts and sub-districts. We also included additional records found at district/sub-district levels, if any, in the final line-list. We conducted sensitization meetings at districts and sub-districts levels with key stakeholders including Deputy Chief Medical Officer of Health-II, Block Medical Officer of Health, Vector Borne Disease Consultant, District Epidemiologist, Block Public Health Nurse, Auxiliary Nurse Midwife, Accredited Social Health Activist and Kala-azar Technical Supervisor. We created separate groups using social messaging application to networking all stakeholders at the sub-district level. Study teams accompanied by Accredited Social Health Activist visited door-to-door and examined all available enlisted individuals for any signs and symptoms of VL/post kala-azar dermal leishmaniasis and tested serologically by rK39 rapid diagnostic test (InBios INC, USA) for the detection of anti-leishmanial antibody. The clinical experts examined all rK39 positive cases with signs and symptoms of post kala-azar dermal leishmaniasis in medical camps for case confirmation. We examined all suspected recurrent post kala-azar dermal leishmaniasis patients parasitologically by microscopy and PCR.

**Fig. 2:**
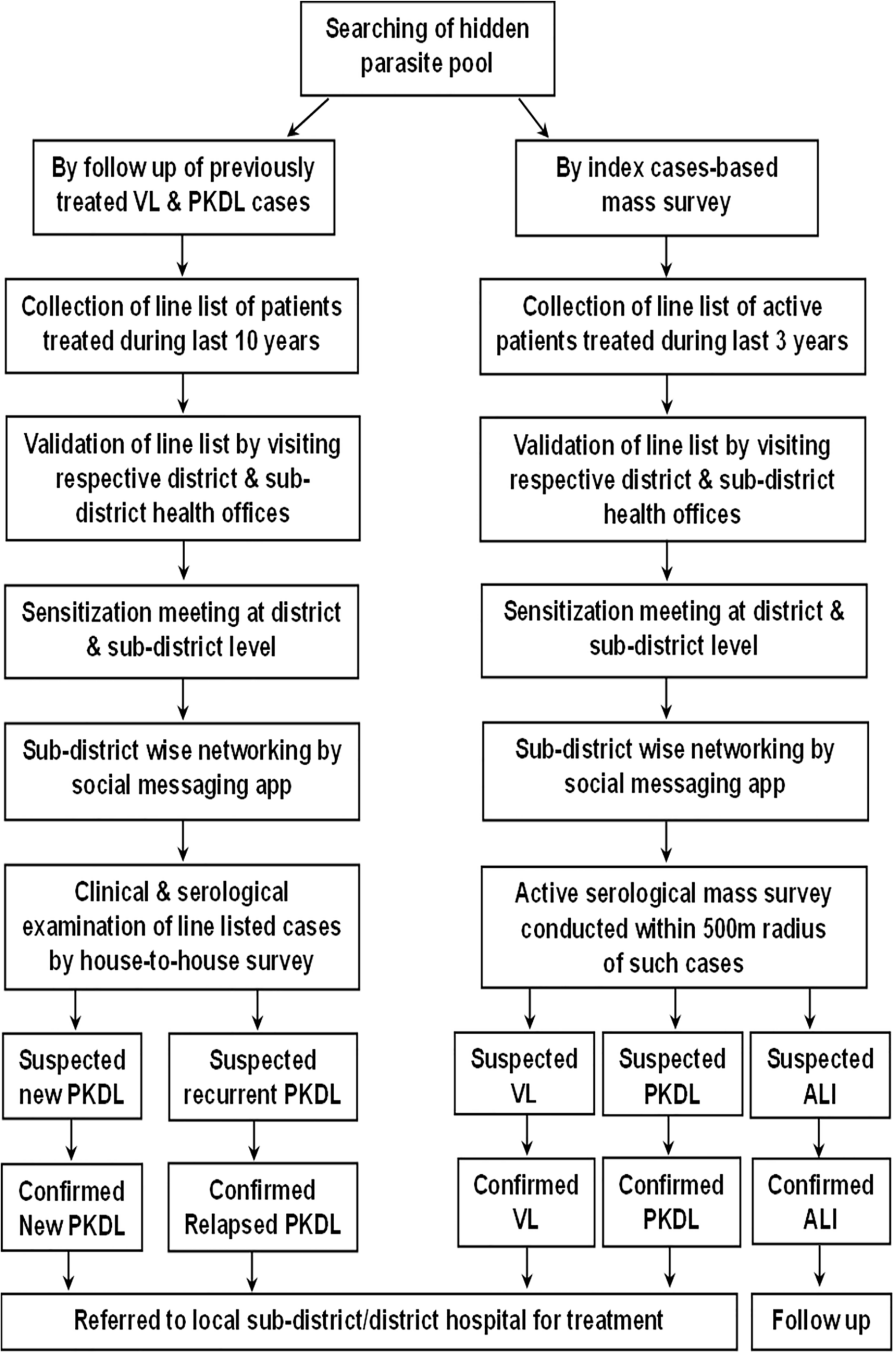
Flow chart showing the study design and searching of hidden pathogen pool. *PKDL* post kala-azar dermal leishmaniasis, *ALI* asymptomatic leishmanial infection

Second, we conducted index case-based mass sero-surveillance around 500 m radius of an active VL/post kala-azar dermal leishmaniasis cases or those treated during previous three years to identify the cases of post kala-azar dermal leishmaniasis, VL and asymptomatic leishmanial infection. Expert clinicians examined all rK39 positive individuals clinically and/or parasitologically, for case confirmation. We followed up the individuals with asymptomatic leishmanial infection clinically and serologically at an interval of 6 months (Fig. 2).

### Case Definition

*New post kala-azar dermal leishmaniasis*: Individuals positive for rK39 test with or without history of VL along with typical signs and symptoms of post kala-azar dermal leishmaniasis were diagnosed as ‘new’ post kala-azar dermal leishmaniasis.

*Recurrent post kala-azar dermal leishmaniasis*: It is defined as ‘an anti-leishmanial antibody positive case having documented history of diagnosis and treatment completion for post kala-azar dermal leishmaniasis with disappearance or significant reduction in dermal lesions and reappearance of new lesions and/ or increase in size of pre-existing residual hypo-pigmented lesions along with parasitological confirmation either by microscopy and/or PCR.

*Asymptomatic leishmanial infection*: It is defined as someone from an endemic area that exhibits an immune response (either humoral or T-cell mediated) against Leishmania or has parasites or parasitic DNA in the blood but remains healthy.

### Collection of Slit Skin Scraping Samples from Post Kala-Azar Dermal Leishmaniasis Patients

We collected the slit skin scraping samples from suspected recurrent post kala-azar dermal leishmaniasis for detection of parasites by microscopy and leishmanial DNA by PCR following proper aseptic conditions. We used one part of tissue fluid and cells for preparation of 2 smears and another part collected in NET buffer [150 mM NaCl, 15 mM Tris–HCl (pH-8.30), 1 mM EDTA] for isolation of DNA.

### Laboratory Investigations

#### Staining of Slit Skin Smear and Microscopy

We stained one smear with Giemsa and other with modified Ziehl–Neelsen stain. Two expert microscopists examined Giemsa-stained smear for LD bodies and Ziehl–Neelsen stained smear to rule out presence of acid-fast bacilli.

#### Isolation of DNA from Slit Skin Scraping Samples for the Detection of Leishmanial DNA by PCR Method

We extracted DNA from the slit skin scraping samples collected in NET buffer by using QIAamp DNA blood mini kit (Qiagen, Hilden, Germany) as per the manufacturer’s instructions. We performed leishmania-specific nested PCR targeting the parasites’ SSU-rRNA region as described by Salam et al. [26]. For each set of PCR reaction, we used DNA from known VL cases as positive control while sterile sigma water as negative control.

### Treatment of VL, New and Recurrent Post Kala-Azar Dermal Leishmaniasis

Local district/sub-district hospitals treated all diagnosed VL and post kala-azar dermal leishmaniasis cases according to the Indian national guidelines. After ruling out HIV co-infection, medical professionals administered a single intravenous infusion of liposomal amphotericin B (10 mg/Kg body weight) to treat VL cases. New and recurrent post kala-azar dermal leishmaniasis patients were treated with oral drug miltefosine for 12 weeks.

### Ethics Statement

Before initiation of survey, study team explained the objectives and benefits to the enlisted patients, head of the family and other members. They were also informed about non-disclosure of their identity and the liberty to withdraw from the study at any time. We obtained written informed consent from suspected recurrent PKDL patients or from legal guardians (for minor) for collection of slit-skin scrapings and verbal consent from the head of the family during mass screening. The Clinical Research Ethics Committee of School of Tropical Medicine, Kolkata has approved the study protocol.

## Results

### Study Subjects

We obtained a line-list of 5145 previously treated VL and post kala-azar dermal leishmaniasis from state health authority and verified with the available data of respective districts and sub-districts. Of them, 2978 (57∙9%) were male and 2167 (42∙1%) were female. During the door-to-door follow-up, we could not identify and traced 379 cases, 598 had died before initiation of the study. Therefore, we targeted a total of 4168 individuals for follow-up.

### Year Wise Incidence of Previously Treated VL and Post Kala-Azar Dermal Leishmaniasis Cases and their Treatment History as Recorded

**Fig. 3:**
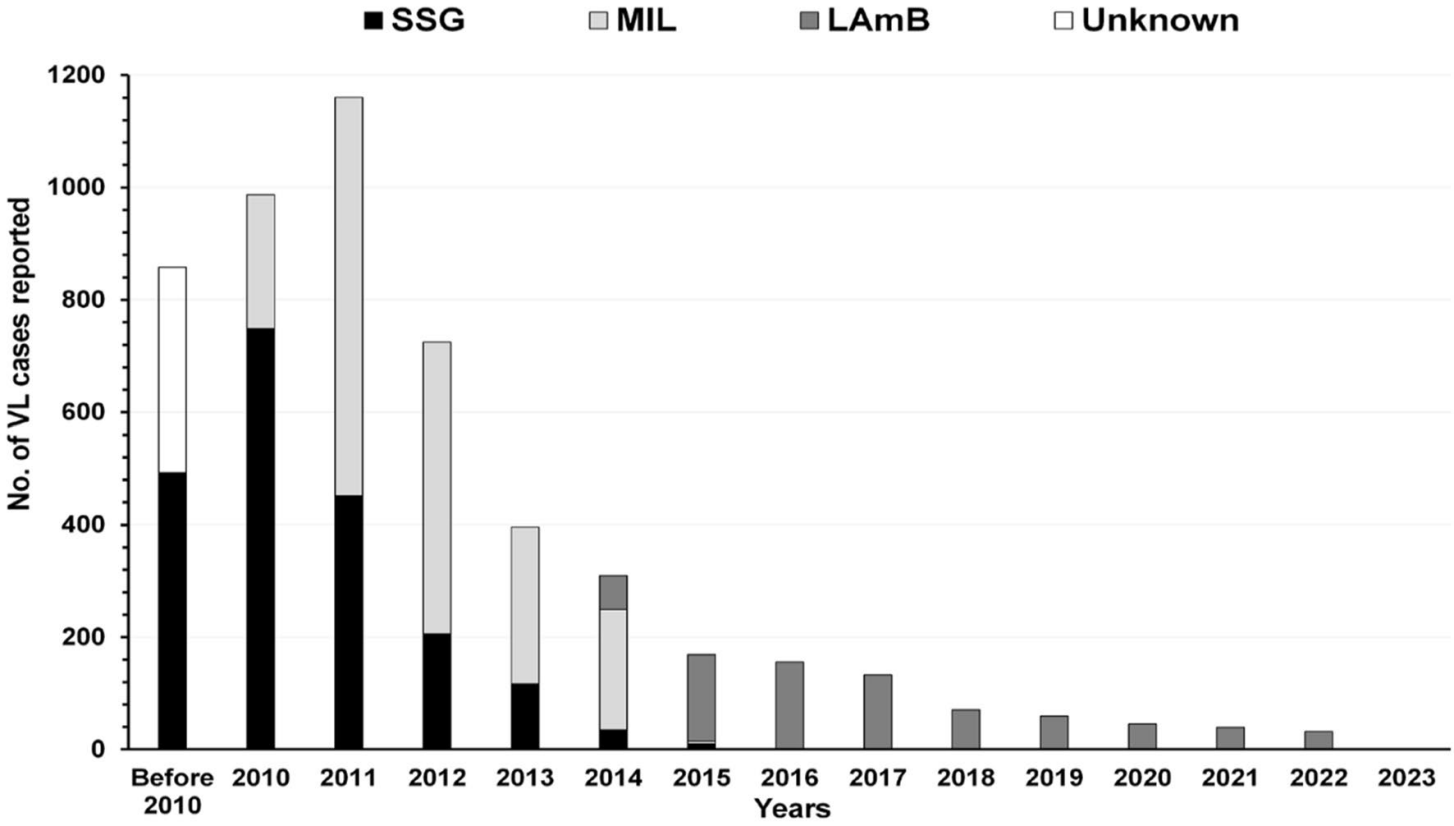
Year wise incidence of VL and their treatment as recorded from the line list of previously treated cases of four study districts of West Bengal, India. *SSG* sodium stibogluconate, *MIL* miltefosine, *LAmB* liposomal amphotericin B

Out of 5145, 858 VL cases were treated before 2010 and 4287 during 2010–2021. We could not ascertain the treatment history of 365 VL cases. Among 4780 patients, 2062 (43.1%), 1969 (41.2%) and 749 (15.7%) were treated with sodium stibo-gluconate, miltefosine and liposomal amphotericin B respectively (Fig. 3).

**Fig. 4:**
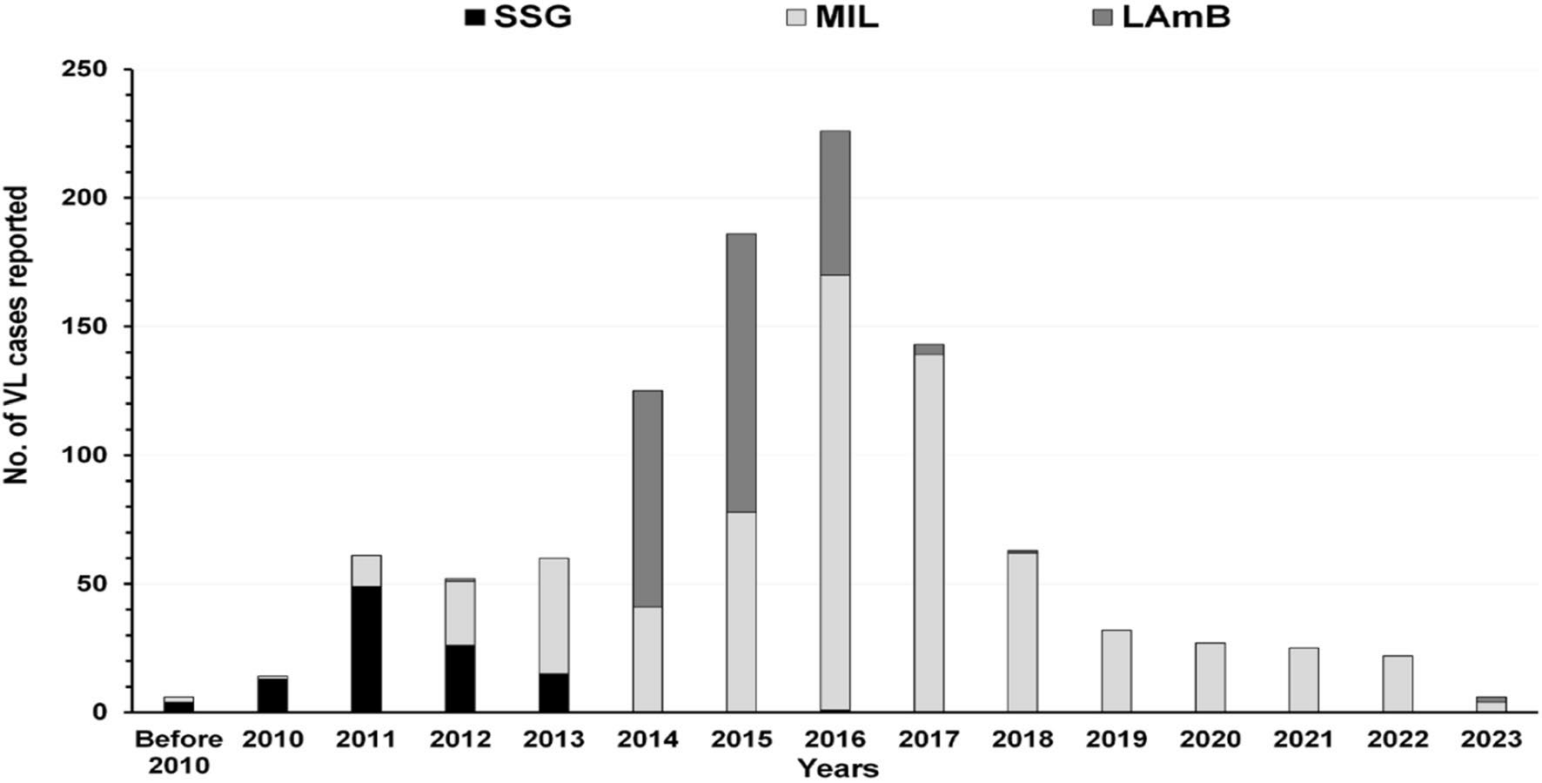
Year wise incidence of post kala-azar dermal leishmaniasis and their treatment as recorded from the line list of previously treated cases of four study districts of West Bengal, India. *SSG* sodium stibogluconate, *MIL* miltefosine, *LAmB* liposomal amphotericin B

**Fig. 5:**
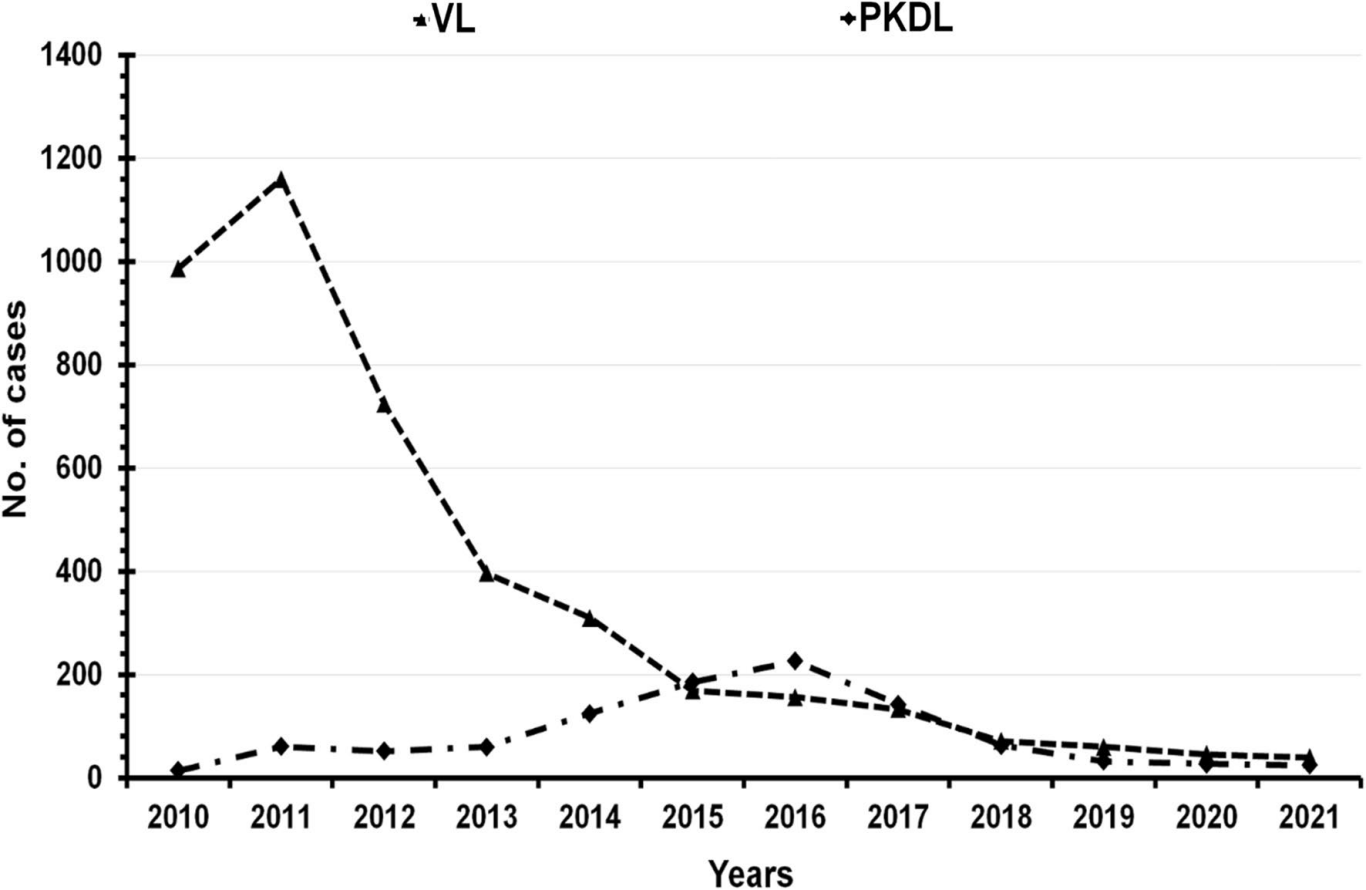
Incidence of previously treated VL and post kala-azar dermal leishmaniasis cases during 2010–2021 in four study districts of West Bengal, India

Among 5145 enlisted patients, 1048 had history of post kala-azar dermal leishmaniasis following VL. Of them 108 (10.3%), 684 (65.3%) and 256 (24.4%) received sodium stibo-gluconate, miltefosine and liposomal amphotericin B respectively (Fig. 4). Incidence of VL was highest in 2011 and that of post kala-azar dermal leishmaniasis in 2016. Thereafter, the annual incidence of both VL and post kala-azar dermal leishmaniasis declined gradually (Fig. 5).

### History of Recurrent VL and Post Kala-Azar Dermal Leishmaniasis as Recorded Among Previously Treated Cases

It was evident from the records that certain number of VL and post kala-azar dermal leishmaniasis cases received treatment more than once. So, they were considered as recurrent VL or post kala-azar dermal leishmaniasis respectively. We recorded 25 recurrent VL, among them 9 (0.44%, 95% CI 0.20–0.83), 13 (0.66%, 95% CI 0.35–1.13) and 3 (0.40%, 95% CI 0.08–1.17) had history of treatment with sodium stibo-gluconate, miltefosine and liposomal amphotericin B respectively for their initial episode of VL (Table 1). Fifty cases had history of recurrent post kala-azar dermal leishmaniasis. Among them, 15 (13.89%, 95% CI 7.99–21.87) were treated with sodium stibo-gluconate, 26 (3.80%, 95% CI 2.50–5.52) with miltefosine and 9 (3.52%, 95% CI 1.62–6.57) with liposomal amphotericin B for their initial episode of post kala-azar dermal leishmaniasis (Table 2).

**Table 1:** Pattern of recurrent VL following treatment with different drugs as recorded from the line list of previously treated cases of four study districts of West Bengal, India

Initial VL treated with	No. of VL cases	No. of recurrent VL	%	95% CI
SSG	2062	9	0.44	0.20–0.83
MIL	1969	13	0.66	0.35–1.13
LAmB	749	3	0.40	0.08–1.17
Unknown	365	0	0	0–1.01
Total	5145	25	0.49	0.31–0.72

*SSG* sodium stibogluconate, *MIL* miltefosine, *LAmB* liposomal amphotericin B

**Table 2:** Pattern of recurrent post kala-azar dermal leishmaniasis (PKDL) following treatment with different drugs as recorded from the line list of previously treated cases of four study districts of West Bengal, India

Initial PKDL treated with	No. of PKDL cases	No. of recurrent PKDL	%	95% CI
SSG	108	15	13.89	7.99–21.87
MIL	684	26	3.80	2.50–5.52
LAmB	256	9	3.52	1.62–6.57
Total	1048	50	4.77	3.56–6.24

*SSG* sodium stibogluconate, *MIL* miltefosine, *LAmB* liposomal amphotericin B

### Incidence of New and Recurrent Post Kala-Azar Dermal Leishmaniasis Among Previously Treated Individuals

Out of 4168 previously treated cases, 3192 (76.6%) had history of VL only and 976 (23.4%) had both VL and post kala-azar dermal leishmaniasis history. We diagnosed 42 (1.9%, 95% CI 1∙4–2.5) as new post kala-azar dermal leishmaniasis among 2248 (70.4%) previously treated VL cases followed up. Out of 42 new post kala-azar dermal leishmaniasis cases, 30 (71.4%) had hypo-pigmented lesions and 12 (28.6%) had mix of hypo-pigmented and papulo-nodular lesions. Out of these 42 diagnosed ‘new’ post kala-azar dermal leishmaniasis, 23 (3.1%, 95% CI 1.9–4.7), 10 (0.9%, 95% CI 0.5–1.8) and 9 (1∙8%, 95% CI 0.8–3.4) had history of sodium stibo-gluconate, miltefosine and liposomal amphotericin B treatment respectively for their initial VL. We observed a significant difference between the incidence of new post kala-azar dermal leishmaniasis cases among sodium stibo-gluconate and miltefosine treated VL cases (p = 0.0021) but no such difference was observed between the incidence of new post kala-azar dermal leishmaniasis cases among miltefosine and liposomal amphotericin B (p = 0.2237) and sodium stibo-gluconate and liposomal amphotericin B (p = 0.2011) treated VL cases. The average interval between initial VL and diagnosis of post kala-azar dermal leishmaniasis was 4∙1 years with a range of 3–17 years.

**Fig. 6:**
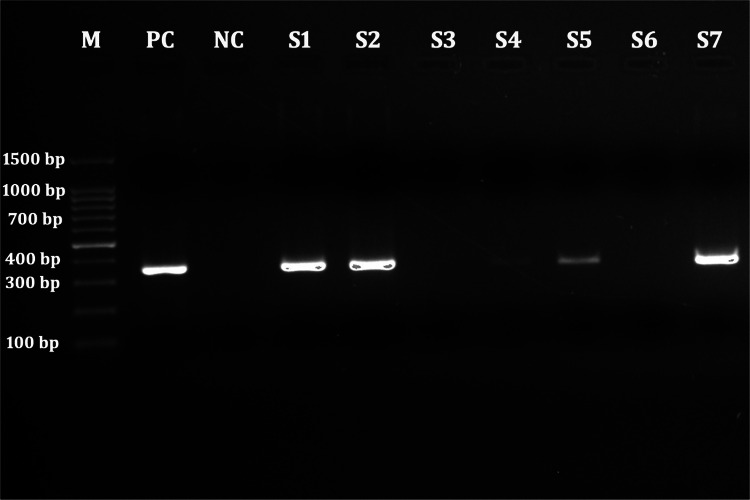
Gel photograph showing the positive and negative PCR reaction for detection of leishmanial DNA among suspected recurrent post kala-azar dermal leishmaniasis patients (*M*  DNA ladder, *PC* positive control, *NC* negative control, *S1-S7* samples)

We confirmed 38 (4.9%, 95% CI 3.5–6.6) recurrent post kala-azar dermal leishmaniasis among 778 (79.7%) cases with history of both VL and post kala-azar dermal leishmaniasis. Among them, parasite was demonstrated in 35 (92.1%) whereas parasitic DNA was detected in 36 (94.7%) cases by PCR (Fig. 6). Eight (21.1%) of them had hypo-pigmented lesions and 30 (78.9%) had mix of hypo-pigmented and papulo-nodular lesions. The treatment history for their initial episode of the disease was 3 (4%, 95% CI 0.8–11.3) with sodium stibo-gluconate, 26 (5.1%, 95% CI 3.4–7.4) with miltefosine and 9 (4.7%, 95% CI 2.2–8.7) with liposomal amphotericin B. There was no significant difference between the incidence of recurrent post kala-azar dermal leishmaniasis across all three previous treatment regimens (p = 1.00) (Table 3). The average interval between initial episode and diagnosis of recurrent post kala-azar dermal leishmaniasis was 1.9 years with a range of 1–9 years. Interestingly 7 cases had more than one episode of recurrent post kala-azar dermal leishmaniasis with a highest of 4 episodes of recurrence in one patient.

**Table 3:** Result of follow up of previously treated VL and post kala-azar dermal leishmaniasis (PKDL) cases during study period in four study districts of West Bengal, India

Total patients pool (n = 5145)	No. of patients could not be identified and traced (n = 379) No. of patients died before initiation of study (n = 598)	Target patients pool (n = 4168)	Total no. of cases with H/O of VL only (n = 3192)	Initial VL treated with	No. of cases	No. of cases followed up clinically & serologically (%)	No. of confirmed new PKDL	% (95% CI)
				SSG	1099	736 (66.9)	23	3.1 (1.9–4.7)
				MIL	1467	1008 (68.7)	10	0.9 (0.5–1.8)
				LAmB	626	504 (80.5)	9	1.8 (0.8–3.4)
				TOTAL	3192	2248 (70.4)	42	1.9 (1.4–2.5)
			Total no. of cases with H/O both VL & PKDL (n = 976)	Initial PKDL treated with	No. of cases	No. of cases followed up clinically & serologically (%)	No. of confirmed recurrent PKDL	% (95% CI)
				SSG	100	75 (75.0)	3	4.0 (0.8–11.3)
				MIL	639	510 (79.8)	26	5.1 (3.4–7.4)
				LAmB	237	193 (81.4)	9	4.7 (2∙2–8∙7)
				TOTAL	976	778 (79.7)	38	4.9 (3.5–6.6)

*SSG* sodium stibogluconate, *MIL* miltefosine, *LAmB* liposomal amphotericin B

### Results of Mass Sero-Surveillance Surrounding 500 m of Active or Treated VL/Post Kala-Azar Dermal Leishmaniasis During Preceding Three Years

We targeted 197 foci for active mass sero-survey with a population of 20,297, of them 9542 (47.01%) were examined. By this way, we diagnosed 8 (0.08%, 95% CI 0.04–0.17) VL and 1 (0.01%, 95% CI 0–0.06) post kala-azar dermal leishmaniasis (without history of VL) and 93 (0.97%, 95% CI 0.79–1.19) asymptomatic leishmanial infection. Individuals with asymptomatic leishmanial infection are being followed up for development of VL or post kala-azar dermal leishmaniasis (Table 4).

**Table 4:** Results of active mass survey surrounding 500 m of active or treated VL/post kala-azar dermal leishmaniasis (PKDL) during preceding three years in four study districts of West Bengal, India

District	Total no. of village targeted	Total population targeted	Total no. of individuals tested	No. of VL detected			No. of ALI detected			No. of PKDL detected		
				n	%	95% CI	n	%	95% CI	n	%	95% CI
Dakshin Dinajpur	69	6828	3107	7	0.23	0.09–0.46	27	0.87	0.57–1.26	0	0	0–0.12
Uttar Dinajpur	39	4923	2039	0	0	0–0.18	23	1.13	0.72–1.69	1	0.05	0–0.27
Malda	80	7890	4072	0	0	0–0.09	38	0.93	0.66–1.28	0	0	0–0.09
Darjeeling	9	656	324	1	0.31	0.01–1.71	5	1.54	0.50–3.56	0	0	0–1.13
Total	197	20,297	9542	8	0.08	0.04–0.17	93	0.97	0.79–1.19	1	0.01	0–0.06

*ALI* Asymptomatic leishmanial infection

## Discussion

Our efforts through a three-step process of mapping and follow-up of previously treated cases, mass surveillance around known cases, and regular clinical and serological screening of asymptomatic cases enabled detection of 42 new and 38 recurrent post kala-azar dermal leishmaniasis. A significant number of recurrent post kala-azar dermal leishmaniasis is a matter of concern for VL elimination programme and attention should be paid to their treatment modalities.

The ongoing VL elimination programme in Indian subcontinent is targeting the elimination of the disease VL but not the pathogen [6]. The main components of the programme are: (a) early case detection and complete treatment, (b) integrated vector management, (c) effective disease surveillance, (d) social mobilization and behavioural changes, and (e) operational research [4–6]. Case detection is performed by three different ways; house-to-house survey, camp-based approach and index case-based mass screening depending upon the epidemiological situation [27]. By adopting these approaches, the annual incidence of VL has come down below the elimination target in Indian sub-continent. The persistence of a significant number of undiagnosed cases of post kala-azar dermal leishmaniasis in the community raises the question whether an annual incidence target for this condition should be incorporated into the National VL elimination program.

Previous studies showed that active door-to-door case search for VL, in addition to passive case detection, resulted in reducing the time to diagnosis and risk of transmission [23]. But no such effort has so far been given for the detection of post kala-azar dermal leishmaniasis. Active search for post kala-azar dermal leishmaniasis is a major constrain as they are apparently healthy and do not seek treatment [28, 29]. It is also difficult to reach all individuals living in endemic areas. In India, majority of post kala-azar dermal leishmaniasis developed among treated VL cases [25]. Considering this, the strategy for case searching need to be modified and focused among the individuals having history of VL and/or post kala-azar dermal leishmaniasis. By this approach we diagnosed and treated 42 new post kala-azar dermal leishmaniasis.

Detection of a significant number of recurrent post kala-azar dermal leishmaniasis (38/778, 4.9%) is an important finding of this study which remained overlooked in terms of its magnitude and treatment modalities. Analysis of the secondary programme data also revealed comparable number of recurrent post kala-azar dermal leishmaniasis (50/1048, 4.8%).

Case confirmation of recurrent post kala-azar dermal leishmaniasis is challenging as it requires demonstration of pathogen by microscopy or by PCR or by both along with clinical signs. We confirmed this by both the methods. Collection and examination (microscopy and PCR) of slit skin samples need some technical expertise and instrumental facilities which are not readily available at the field level. Two to three block primary health centres per district may be identified and existing clinical and laboratory staff can to be trained for this purpose.

We did not find any significant association between recurrences of post kala-azar dermal leishmaniasis with any specific anti-leishmanial drug. Presently, liposomal amphotericin B is used for the treatment of VL and miltefosine for post kala-azar dermal leishmaniasis. Miltefosine is provided to the patients on weekly basis for 12 weeks. Since supervised administration for each dose is not feasible, the adherence to miltefosine cannot be ascertained accurately. But recurrence of post kala-azar dermal leishmaniasis following treatment with liposomal amphotericin B is a matter of concern, as it is administered following hospitalization. So, the doses and duration of liposomal amphotericin B for the treatment of post kala-azar dermal leishmaniasis need a relook. There is no guideline for the treatment of recurrent post kala-azar dermal leishmaniasis both at national and international level. So, clinical trials are necessary to explore various dosages and durations of liposomal amphotericin B in combination with miltefosine or other anti-leishmanial drugs for formulation of appropriate guideline. Researchers and policymakers must prioritise this issue.

Majority of treated post kala-azar dermal leishmaniasis patients ignore the reappearance of skin lesions that delayed diagnosis and initiation of treatment. This results in persistence of parasites in the community. Regular awareness among the residents of endemic areas is an important aspect of ongoing programme through information, education, communication and behavioural, change, communication activities. Since the knowledge about the disease is moderate to poor among 75.2% population [30], enhancement of health education in endemic areas will improve the treatment seeking behaviour.

Recently, the asymptomatic leishmanial infection has drawn attention regarding its role in maintaining parasite in the community. Several studies have documented significant number of asymptomatic leishmanial infection in endemic areas including West Bengal [31–33]. Identification of asymptomatic leishmanial infection is only possible by mass sero-surveillance of the entire population by rK39 test which is labour and resource-intensive. Serological screening of population surrounding 500 m of active and recently treated cases is a feasible cost-effective alternative and included in the national policy. We have diagnosed 93 asymptomatic leishmanial infection, 8 VL and 1 post kala-azar dermal leishmaniasis. Among 93 asymptomatic leishmanial infection, disease conversion to VL was recorded in one individual during one year follow-up. The main challenge faced during follow-up was the unavailability of a significant number of previously treated individuals due to migration. Additional measures should be strategized to bring migrant workers under clinic-parasitological surveillance.

Vector control is one of the most important aspects for prevention of resurgence of VL. In India, indoor residual spray of DDT was in practice till 2016. It has been replaced by a synthetic pyrethroid - alpha cypermethrin due to emergence of DDT resistance. The used insecticide is effective but its application need close monitoring [34].

## Conclusion

For elimination of pathogen pool, additional thrust should be given for detection of new and recurrent post kala-azar dermal leishmaniasis through follow-up of all previously treated VL and post kala-azar dermal leishmaniasis cases as it was done in the present study along with existing case search methods. It should be continued for long period as post kala-azar dermal leishmaniasis can develop after many years. Similarly, previously treated post kala-azar dermal leishmaniasis cases should also be observed for recurrence of skin lesions. Similar studies in other VL endemic areas are suggested to ascertain the feasibility and effectiveness of the case finding strategy deployed in this study. A clinical trial with different combination of anti-leishmanial drugs for formulation of treatment policy for recurrent post kala-azar dermal leishmaniasis is also warranted. This may help the policymakers at national and international level to develop appropriate strategy for eliminating pathogen to prevent resurgence of VL in future.

## Data Availability

All relevant data are within the manuscript and its supporting information files i.e., Tables and Figures.

## Abbreviations

VL: Visceral leishmaniasis
PCR: Polymerase Chain Reaction
WHO: World Health Organization
rK-39: Recombinant product of K39
DNA: Deoxyribo nucleic acid
HIV: Human immunodeficiency virus
p value: Probability value
